# Grade 2, 3 and Dedifferentiated Chondrosarcomas: A Comparative Study of Isocitrate Dehydrogenase-Mutant and Wild-Type Tumors with Implications for Prognosis and Therapy

**DOI:** 10.3390/cancers16020247

**Published:** 2024-01-05

**Authors:** Karen Schoedel, Tanya Heim, Anette Duensing, Ines Lohse, Laura Presutti, Rebekah Belayneh, Sumail Bhogal, Anya Singh-Varma, Alexander Chang, Uma Chandran, Daniel Marker, Heather Szabo-Rogers, Kurt Weiss

**Affiliations:** 1 Department of Pathology, University of Pittsburgh, Pittsburgh, PA 15213, USA; 2 Department of Orthopaedic Surgery, University of Pittsburgh, Pittsburgh, PA 15232, USA; 3 School of Medicine, University of Pittsburgh, Pittsburgh, PA 15213, USA; 4 Department of Biomedical Informatics, University of Pittsburgh, Pittsburgh, PA 15206, USA; 5 Department of Anatomy, Physiology and Pharmacology, College of Medicine, University of Saskatchewan, Saskatoon, SK S7N 5E2, Canada

**Keywords:** chondrosarcoma, RNA sequencing, isocitrate dehydrogenase, genomics

## Abstract

**Simple Summary:**

Grade 2 and 3 and dedifferentiated chondrosarcomas represent rare malignant bone neoplasms. These tumors are often associated with isocitrate dehydrogenase (*IDH*) mutations. Unfortunately, treatment options are limited for advanced disease and at the present time both *IDH* mutant and WT tumors are treated similarly. This study compares differential gene expression in *IDH* mutant and WT chondrosarcomas with RNA sequencing and stratifies clinical outcome by *IDH* status and tumor grade.

**Abstract:**

Background: Grade 2 and 3 and dedifferentiated chondrosarcomas (CS) are frequently associated with isocitrate dehydrogenase (*IDH*) mutations and often exhibit a poor clinical outcome. Treatment is limited mainly to surgery. Defining *IDH* status (wild type (WT) and mutant) and the associated transcriptome may prove useful in determining other therapeutic options in these neoplasms. Methods: Formalin-fixed paraffin-embedded material from 69 primary and recurrent grade 2, 3 and dedifferentiated CS was obtained. DNA sequencing for *IDH1* and *IDH2* mutations (*n* = 47) and RNA sequencing via Nextseq 2000 (*n* = 14) were performed. Differentially expressed genes (DEGs) were identified and used to predict aberrant biological pathways with Ingenuity Pathway Analysis (IPA) software (Qiagen). Gene Set Enrichment Analyses (GSEA) using subsets C3, C5 and C7 were performed. Differentially expressed genes were validated by immunohistochemistry. Outcome analysis was performed using the Wilcoxon test. Results: A set of 69 CS (28 females, 41 males), average age 65, distributed among femur, pelvis, humerus, and chest wall were identified from available clinical material. After further selection based on available *IDH* status, we evaluated 15 *IDH* WT and 32 *IDH* mutant tumors as part of this dataset. Out of 15 *IDH* WT tumors, 7 involved the chest wall/scapula, while 1 of 32 mutants arose in the scapula. There were far more genes overexpressed in *IDH* WT tumors compared to *IDH* mutant tumors. Furthermore, *IDH* WT and *IDH* mutant tumors were transcriptomically distinct in the IPA and GSEA, with *IDH* mutant tumors showing increased activity in methylation pathways and endochondral ossification, while *IDH* WT tumors showed more activity in normal matrix development pathways. Validation immunohistochemistry demonstrated expression of WT1 and AR in *IDH* WT tumors, but not in *IDH* mutants. SATB2 was expressed in *IDH* mutant tumors and not in WT tumors. Outcome analysis revealed differences in overall survival between mutant and WT tumors (*p* = 0.04), dedifferentiated mutant and higher-grade (2, 3) mutant tumors (*p* = 0.03), and dedifferentiated mutant and higher-grade (2, 3) WT tumors (*p* = 0.03). The longest survival times were observed in patients with higher-grade WT tumors, while patients with dedifferentiated mutant tumors showed the lowest survival. Generally, patients with *IDH* WT tumors displayed longer survival in both the higher-grade and dedifferentiated groups. Conclusions: Grade 2, 3 and dedifferentiated chondrosarcomas are further characterized by *IDH* status, which in turn informs transcriptomic phenotype and overall survival. The transcriptome is distinct depending on *IDH* status, and implies different treatment targets.

## 1. Introduction

Conventional chondrosarcoma is the second most common primary malignancy of bone [1,2,3,4]. Approximately 50% to 70% of these tumors harbor isocitrate dehydrogenase (*IDH*) *1* or *IDH2* mutations [5,6,7]. *IDH* mutations are thought to represent an early driver event of oncogenesis in conventional chondrosarcoma [5,8].

Clinical outcome is stratified by grade, based on cellularity, increasing nuclear pleomorphism, and mitoses. Dedifferentiated chondrosarcoma (DDCS) is characterized by a biphasic histology; an area with cartilage matrix is juxtaposed to a highly cellular, pleomorphic sarcoma devoid of matrix. Similar mutations are present in both components, supporting a common origin [9]. However, histologic features do not inform *IDH* status in these malignancies. Approximately 10% of conventional chondrosarcomas undergo dedifferentiation and are associated with the worst prognosis [10]. Treatment options are limited because chondrosarcoma is resistant to adjuvant therapies and represents a mainly surgically treated disease [11].

National Comprehensive Cancer Network (NCCN 1.2024) guidelines for chondrosarcoma recommend wide excision with negative margins in surgically resectable cases [12]. However, unresectable, locally advanced, and widely metastatic chondrosarcoma require systemic therapy in an effort to control disease progression. Post-operative radiation therapy may be offered for conventional chondrosarcomas in select situations. Dedifferentiated chondrosarcoma may be treated with cytotoxic drug regimens similar to those used in osteosarcoma [12,13]. Conventional chondrosarcoma is typically resistant to chemotherapy and there are no uniformly established protocols [12,14]. Other novel therapies include tyrosine kinase inhibition by agents such as dasatinib [15] and pazopanib [16], and *IDH1* inhibition with application of ivosidenib in patients with susceptible mutations [11]. Comprehensive molecular testing may be considered to determine potential treatment targets in individual patients [12].

In this work, we compared the genomic and transcriptomic characteristics of *IDH* mutant to wild type dedifferentiated and higher-grade (2, 3) chondrosarcomas. The results obtained may impact potential therapeutic options for these aggressive neoplasms.

## 2. Methods

### 2.1. Case Material

Formalin-fixed paraffin-embedded (FFPE) material from 69 primary and recurrent conventional higher-grade (2, 3) and dedifferentiated chondrosarcomas from 1999–2021 was obtained from the UPMC Department of Pathology under IRB approval (IRB 20050109). Hematoxylin and eosin slides were reviewed by a senior musculoskeletal pathologist (KS) and regions of interest were selected for tissue microarray construction (2 mm cores).

### 2.2. IDH Analysis

DNA was extracted from FFPE material and targeted amplification Sanger sequencing was performed for *IDH1* and *IDH2* mutations using ampliTAQ Gold360 PCR Master Mix (Applied Biosystems, Waltham, MA, USA) and capillary gel electrophoresis on the ABI3730xl (Applied Biosystems) [17].

Interpretable results were obtained in 47 cases.

### 2.3. RNA Sequencing

RNA was extracted from FFPE material (8 *IDH*-mutant, 6 *IDH* wild-type) after quality control (Tape Station HSD1000). Samples were sequenced via Nextseq 2000. The reverse-stranded paired-end RNA-Seq reads were checked for the presence of adapters and high-quality bases using FastQC (v 0.11.7). These high-quality reads were trimmed for the TruSeq adapters using Cutadapt (v 1.18). The trimmed reads were later mapped against the Ensembl human reference genome (GRCh38 v 107) using the STAR (v 2.7.9a) mapping tool. For better mapping outcomes, the STAR parameters were modified to utilize outFilterScoreMinOverLread and outFilterMatchNminOverLread, where both parameters were set to 0.3 instead of the standard. The output file from STAR was converted from SAM format to BAM format using SAMtools (v 1.9). Counts for expressed genes were generated using HT-Seq (v 0.11.2) and output was generated in text format. These count text files were then imported into the Bioconductor R package, edgeR (v 3.38.4). After importing the counts text files, ComBat seq was performed from the sva package (v 3.44.0) to compensate for the different sequencing batches without removing biological differences between samples. The edgeR package was then again utilized to identify differentially expressed genes based on the criteria of the genes having an expression count of absolute value log base 2 greater than 1 between two experimental conditions and a false discovery rate of less than 0.05 using an Exact test. Based on this standard, a single comparison of 6 wild type vs. 8 *IDH* mutant samples produced 743 differentially expressed genes.

After the differentially expressed genes were identified each list of genes along with their differential expression values were uploaded to Ingenuity Pathway Analysis (IPA) and used to identify aberrant biological pathways (FDR 0.05). Gene Set Enrichment Analyses (GSEA) were performed using the GSEA software (v 4.2.1 [build 5]) from the Broad Institute. Subsets C3, C5 and C7 were utilized in the GSEA. Immunohistochemistry: Differentially expressed genes (Wilms Tumor 1, *WT1*, Androgen Receptor, *AR)* and (Special AT-rich sequence-binding protein 2, *SATB2* suggested by the GSEA) were validated on tissue microarrays (TMAs), and whole tumor sections using monoclonal antibodies (WT1 predilute, Ventana-Roche Oro, Valley, AZ, USA,; AR 1:100, Dako Santa Clara, CA, USA; SATB2 predilute, Cell Marque, Rocklin, CA, USA).

### 2.4. RT-qPCR Analysis

A subset of 5 *IDH* mutant samples of fresh frozen tissue were analyzed for the presence of SATB2, MMP13, and COL10A1 using commercially available primers. Briefly, RNA was extracted from a subset of 5 *IDH* mutant fresh frozen tissue samples according to the manufacturer’s protocol (Qiagen RNeasy Mini Kit, 74106 Germantown, MD, USA). RNA was analyzed using a one-step RT-qPCR protocol (Bio-Rad iTaq Universal SYBR Green One-Step Kit, 1725151) at a total reaction volume of 20 µL. GAPDH and SYMPK were used as housekeeping gene references. Each reaction was setup according to the manufacturer’s protocol using 150 ng RNA input for each respective sample reaction. Reactions were set up in a 384-well hard-shell plate and loaded on a CFX Opus 384 RT-qPCR instrument (Bio-Rad, Cat #12011452 Hercules, CA, USA). Expression data were analyzed using CFX Maestro Software (version 2.3).

### 2.5. Outcome Analysis

Clinical outcome data were obtained from UPMC electronic medical records and the UPMC Network Cancer Registry. Kaplan–Meier plots were generated and statistical analyses were performed using Wilcoxon tests and Prism9.

## 3. Results

### 3.1. Patients

Sixty-nine primary and recurrent conventional higher-grade (2, 3) and dedifferentiated conventional chondrosarcomas from 28 females and 41 males, average age 65 (range 14–91), were collected from the UPMC Department of Pathology archives. The primary sites included mainly the femur, pelvis, humerus, and chest wall (Table 1). All patients were treated surgically. Histologically, the dedifferentiated chondrosarcomas appeared similar to each other with an absent chondroid matrix, spindled-to-epithelioid and occasionally rhabdoid cells, mitotic activity, and necrosis.

### 3.2. IDH Analysis

DNA analysis of FFPE tissue from 47 high-grade and dedifferentiated chondrosarcomas revealed 15 WT and 32 mutant tumors. Of the mutants, 20 were *IDH1,* and 12 were *IDH2*. Correlation with the site of disease demonstrated chest wall/scapula involvement in 7 of 15 WT tumors, while only 1 of 32 mutant tumors was located in the scapula, and none in the chest wall (Table 1).

### 3.3. RNA Sequencing

**Figure 1:**
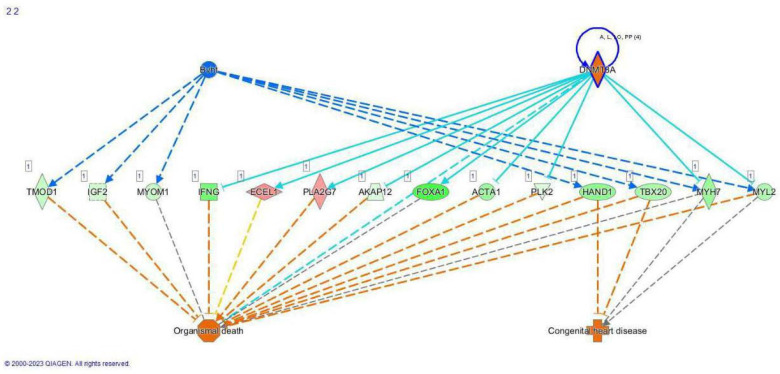
Qiagen IPA diagram demonstrating differences in gene expression between *IDH* WT (indicated by BvHT in blue) and *IDH* mutant (indicated by DNA methyltransferase 3 alpha, DNMT3A in an orange diamond rimmed by purple) tumors. Implicated pathways are generated by Qiagen IPA software based on DEG data input and suggest that endothelin-converting enzyme-like 1 (ECEL1) and phospholipase A2 group VII (PLA2G7) may mediate cell death (solid arrows) in the *IDH* mutant tumors. Dashed lines are suggested pathways.

Differentially expressed genes were entered into the Qiagen IPA to predict associated pathways (IPA, FDR 0.05) and showed that *IDH* WT and mutant tumors were transcriptomically distinct (Figure 1). Furthermore, *IDH* mutant tumors were associated with DNA methyltransferase pathways. Genes implicated in malignant behavior included increased expression of collagen 10 alpha 1 (*COL10A1*), matrix metalloproteinase 13 (*MMP13*), *TP53,* and *WRAP53*. By contrast, the IPA implicated long non-coding RNAs such as Braveheart (*BvHT*) in WT tumors. RNA-sequencing exhibited higher differentially expressed genes (DEGs) in WT tumors than in the mutants. Several development-associated genes such as SRY-box 2 (*SOX2*), insulin-like growth factor-2 (*IGF2*) and the Homeobox family of genes revealed increased expression in the WT tumors. The data were also interrogated via GSEA using subsets C3, C5 and C7. Selected differentially expressed genes (DEGs) correlating with pathways highlighted in the IPA are listed in Table 2. The complete lists of DEGs are included in the Supplementary Materials (Data S1 and S2).

**Figure 2:**
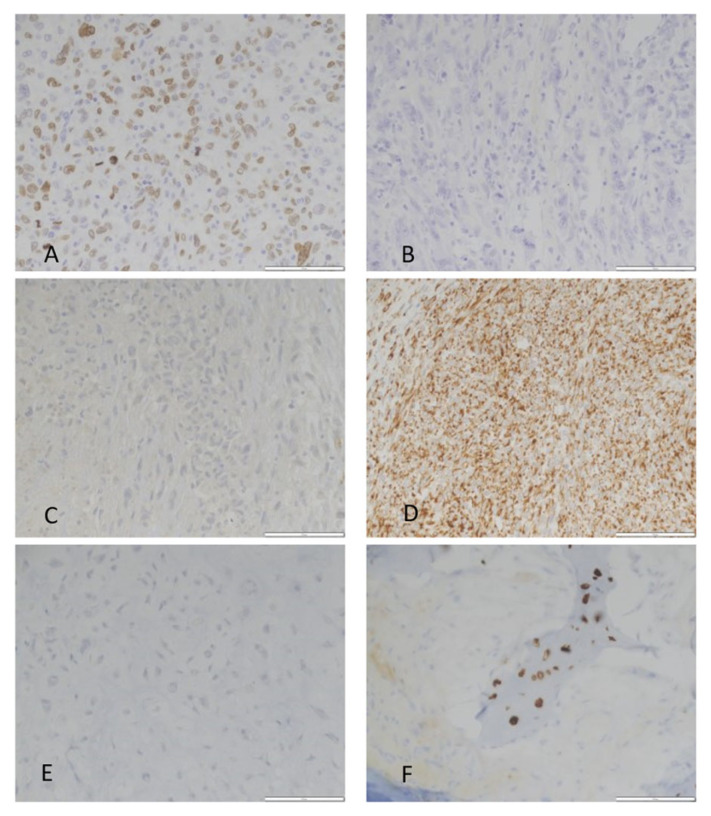
Immunohistochemical validation of SATB2, WT1 and AR in *IDH* mutant and wild type chondrosarcoma: (**A**) SATB2 shows nuclear positivity in *IDH* mutant CS. (**B**) SATB2 immunostaining is negative in WT CS. (**C**) WT1 immunostaining is negative in dedifferentiated *IDH* mutant CS; (**D**) WT1 cytoplasmic and focal nuclear positivity in an *IDH* WT dedifferentiated CS; (**E**) AR negative *IDH* mutant CS; and (**F**) AR positive *IDH* WT CS. All photomicrographs 400×.

**Figure 3:**
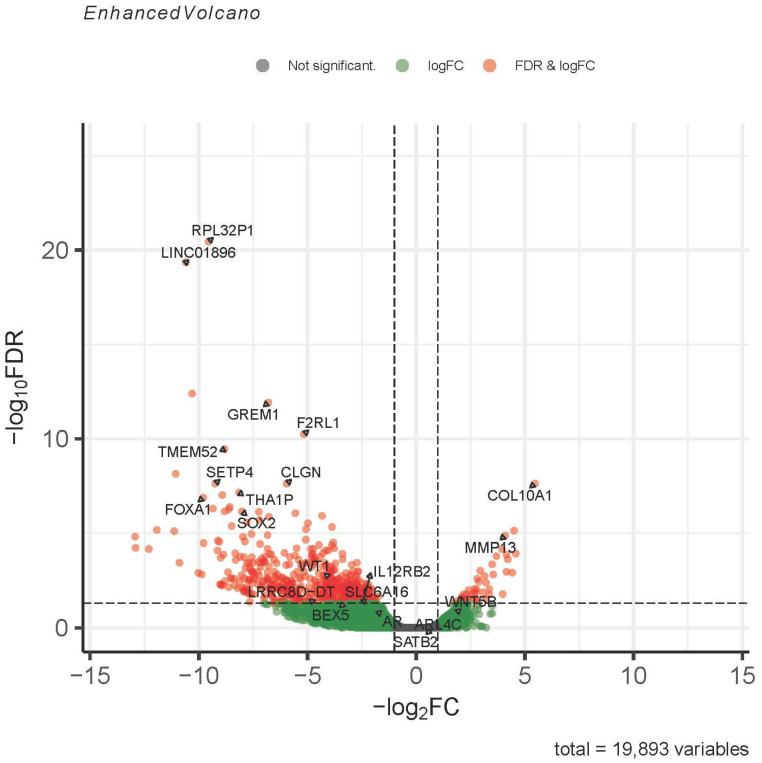
Volcano plot comparing gene expression in *IDH* mutant/WT CHS. Expression of development-related genes are decreased in *IDH* mutant CHS compared to wild type whereas transcripts related to endochondral ossification are increased in the *IDH* mutant population compared to WT. Transcripts highlighted in red pass abs log2FC > 1 and FDR < 0.05; genes in the green zone pass only abs log2FC > 1; and transcripts highlighted in grey zone do not meet either FC or FDR thresholds. The gene expression used for validation by immunohistochemistry includes *WT1* (red zone), *AR* (green zone) and *SATB2* (grey zone). *SATB2* was highlighted by a GSEA gene set (C3) in the *IDH* mutant tumors (see Supplementary Materials Data S3).

Immunohistochemical stains were performed for WT1, AR, and SATB2. Immunohistochemical stains were chosen based on gene expression or highlighted in the GSEA and ease of interpretation (nuclear staining). *WT1* and *AR* were associated with the WT tumors. Although SATB2 did not meet defined cutoffs for a differentially expressed gene, it was identified in the GSEA and immunohistochemistry revealed positivity only in the mutant tumors (Figure 2 and Figure 3 and Supplementary Materials Data S3). Additionally, a subset of *IDH* mutant tumors was subjected to RT-qPCR testing for MMP13, Col10A1 and SATB2 expression (Table 3).

**Figure 4:**
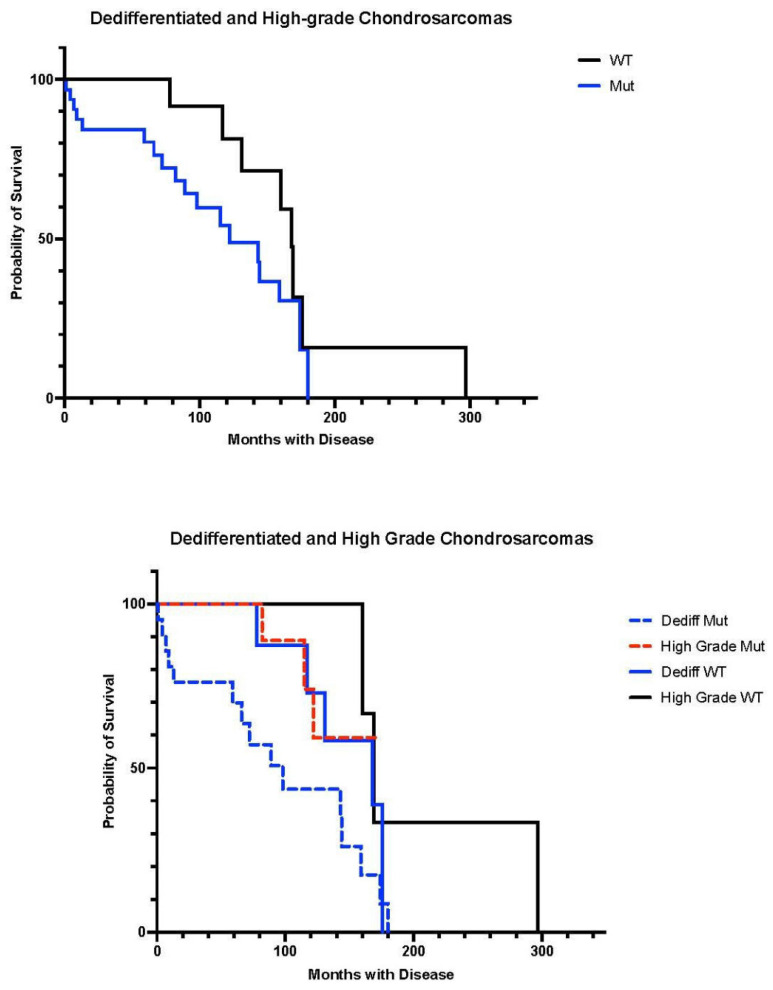
Kaplan–Meier plots of *IDH* mutant (both *IDH1* and *IDH2*) vs. WT overall survival in dedifferentiated and high-grade (2, 3) chondrosarcomas. Top plot shows WT vs. mutant tumors, while bottom plot compares dedifferentiated and high-grade conventional CS stratified by *IDH* status.

Outcome analysis was performed and Kaplan–Meier plots were generated (Figure 4). The difference in survival between *IDH* mutant and WT tumors was statistically significant (*p* = 0.04). Statistically significant differences were also seen between dedifferentiated mutant and high-grade mutant (*p* = 0.03), and dedifferentiated mutant and high-grade WT tumors (*p* = 0.03) In general, wild-type tumors showed a survival advantage.

## 4. Discussion

Histologic grading of conventional chondrosarcoma is correlated with outcome, with high-grade tumors demonstrating greater metastatic potential and mortality than lower-grade disease [1,5]. Dedifferentiated chondrosarcomas (DDCS) are thought to derive from conventional chondrosarcoma, and rarely from enchondroma or osteochondroma [10], and represent a clinically aggressive form of chondrosarcoma. DDCS is associated with a 7–24% 5-year survival [1]. In the majority of cases, treatment is limited mainly to surgery, leaving those with advanced disease without effective therapeutic options. In order to develop new therapies for higher-grade and dedifferentiated CS, the genomic and transcriptomic landscape of these tumors must be understood.

It is known that *IDH* status plays important prognostic and therapeutic roles in several malignancies such as acute myeloid leukemia, cholangiocarcinoma, and glioma [18], among others. In the central nervous system, *IDH* mutant infiltrating gliomas (oligodendroglioma and *IDH* mutant astrocytoma) show significantly longer survival and better clinical outcomes than their *IDH* wild type counterparts (glioblastoma, *IDH* wild type) [19]. The clinical outcomes are so divergent that *IDH* mutational status now defines these diagnostic entities in the most recent WHO classifications [20]. It is interesting to note that in the current study, *IDH* mutational status has the opposite prognostic impact in chondrosarcoma. *IDH* mutant gliomas additionally have distinct morphology and co-occurring molecular alterations; 1p/19q-codeletion and TERT promoter mutation in oligodendroglioma and p53/ATRX mutations in *IDH* mutant astrocytoma. By contrast, *IDH* mutant and WT chondrosarcomas are histologically identical, and at present are treated as a single disease entity.

*IDH* mutations affect 38–86% of conventional chondrosarcomas [21]. *IDH* status appeared to inform the prognosis of higher-grade CS and DDCS in our series. *IDH* WT cases demonstrated prolonged survival in comparison to *IDH* mutants. That *IDH* mutations in chondrosarcoma confer a worse outcome was also seen in Nakegawa’s study of 38 cases in 2022 [2]. In our study, *IDH1* and *IDH2* mutant cases were combined for outcome analysis due to a small number of *IDH2* mutant chondrosarcomas in our series. This is commensurate with the literature, as it is known that chondrosarcomas are affected more often by *IDH1* mutations than *IDH2* [5]. Differentially expressed genes derived from RNA sequencing delineate differences between the *IDH* mutant and WT chondrosarcoma cohorts. They suggest that the molecular pathways utilized for tumor growth, maintenance, and malignant phenotype could be different between the two groups. In a study of 350 cases of chondrosarcoma by Cross et al., it was proposed that *IDH* mutant and wild type tumors utilized different molecular pathways and, furthermore, that *IDH2* mutant and high-grade chondrosarcomas were more often associated with TERT mutations [5]. TERT mutations were also identified in approximately 35% of dedifferentiated chondrosarcomas in Nacev’s study [22]. We did not assess for TERT promoter mutations in our cohort.

Mutations in *IDH1/2* are thought to be oncogenic through the aberrant production of D2-hydroxyglutarate (D2-HG) [7]. D2-HG is an oncometabolite that leads to DNA and histone hypermethylation and associated genome-wide alterations in gene expression [23]. Hypermethylation of *IDH* mutant chondrosarcomas activates proliferation and glycolysis [24], and is associated with higher histologic grade [25]. Amary et al. reported that D2-HG levels were elevated in patients with *IDH* mutated chondrosarcomas arising in the setting of Ollier and Maffucci syndromes [26], and Mohammad et al. showed increased 2-HG levels due to *IDH1* mutations in chondrosarcoma [27].

*IDH* mutant chondrosarcoma demonstrated increased expression of collagen 10 alpha 1 (*COL10A1*) and matrix metalloproteinase 13 (*MMP13*) in our study. The elevated *MMP13* expression suggested that extracellular matrix breakdown may promote aggressive behavior [28]. Vascular invasion may also be facilitated by *MMP13* [29]. Furthermore, *COL10A1* and *MMP13* are involved in endochondral ossification, a process conserved in matrix-producing chondrosarcoma, and downregulated in dedifferentiated chondrosarcoma [24]. The Hedgehog pathway was also implicated in our study, by higher expression of *HHIP* (Table 2). Hedgehog signaling was noted in a study of chondrosarcomas by Isenlys et al., and Tiet et al. showed increased expression of *PTCH1* and *GlI1*, target genes of the Hedgehog pathway [30,31]. *TP53* showed increased expression in the mutant tumors. *TP53* overexpression has been previously reported in chondrosarcoma by several authors [2,5,6,22,32].

Additionally, WRAP53 was overexpressed in the *IDH*-mutant tumors, suggesting that the tumor cells may achieve immortality by telomere lengthening [33].

Furthermore, fewer genes showing increased expression were seen in the *IDH* mutant cohort, suggesting loss of genetic material and/or increased chromosomal instability as compared to the WT group. Special AT-rich sequence-binding protein 2 (*SATB2*) was expressed only in the *IDH* mutants by immunohistochemistry, corroborating its presence in the GSEA, and possibly representing post-translational mechanisms for increased expression. Confirmation of *MMP13*, *COL10A1* and *SATB2* was attempted by RT-qPCR in the *IDH* mutant samples, and expression of these three genes was detected in all samples tested (n = 5). Furthermore, higher expression of *MMP13*, *COL10A1* and *SATB2* was found in two *IDH* mutant samples, both demonstrating an *IDH1* R132C mutation, suggesting that a specific *IDH1* mutation involving R132C may play a role in the expression of these genes. However, the case numbers were too small to form a conclusion. This finding would require a multi-center large chondrosarcoma cohort to study.

With regard to the *IDH* WT tumors, more differentially expressed developmental genes and pathways appeared to play roles in their genesis than seen in the mutants. These included SRY-box 2 (*SOX2)*, Bone morphogenetic protein 7 (*BMP7*), Homeobox A2 (*HOXA2*), Gremlin 1 (*GREM1*), Forkhead box protein A1 (*FOXA1*), Transmembrane protein 52 (*TMEM52*), Insulin-like growth factor 2 (*IGF2*), melanoma antigen (*MAGEA1*), and *ALX1* and *ALX3* Homeobox genes. Braveheart (*BvHT*) was implicated by the IPA analysis. These suggested the involvement of long non-coding RNAs in tumor development, as well as in promotion of tumor cell survival and proliferation. Several of these genes participate in normal embryonic development [34,35,36]. Long non-coding RNAs are involved in cardiovascular disease and many cancers [37]. That *MAGEA1* is expressed in the WT tumors and not in the mutants suggests that *MAGEA1* is epigenetically silenced in the mutants, possibly by DNA methyltransferase 1 (*DNMT1)* and histone deacetylation [38]. *MAGEA1* expression may contribute to malignancy in the WT tumors. WT1 and AR expression were identified in the WT cases and were used as immunohistochemical validation markers.

Interestingly, *IDH* WT tumors were identified more often than *IDH* mutant tumors in the chest wall and scapula, a finding also reported by Cross et al. [5]. This finding may be related to reduced endochondral ossification involved in chest wall development as compared to the long bones. This hypothesis requires further investigation.

Incorporating differential gene expression in *IDH* WT and mutant chondrosarcomas to inform present and future treatment strategies represents a substantial and potentially exciting challenge. With respect to current systemic therapy, while dedifferentiated chondrosarcoma is often treated along an osteosarcoma paradigm using cytotoxic drugs [12,13], chemotherapy in conventional chondrosarcoma has poor activity [14]. However, tyrosine kinase inhibition by dasatinib [15] or pazopanib [16] may offer benefit in terms of disease control and occasional responses in conventional chondrosarcoma. A phase I trial demonstrated the promising activity of an *IDH1* inhibitor, ivosidenib, particularly in conventional chondrosarcoma, where the progression-free survival was greater than in dedifferentiated chondrosarcoma, although the study was not powered to specifically compare those groups [11]. Ivosidenib is currently listed on the National Comprehensive Cancer Network (NCCN) guidelines for conventional and dedifferentiated chondrosarcoma patients with susceptible mutations [12].

Based on the RNA-seq data generated as part of this study, anti-methylation drugs such as Decitabine could be explored pre-clinically in *IDH* mutant tumors, as well as inhibitors of *IDH1* [11,18,21] or *IDH2* [8]. ARL4C expression in the *IDH* mutants (Supplementary Materials Data S4) could represent a target for ASO-1316 [39]. For *IDH* WT tumors, Isotretinoin targeting SOX2 may be considered. Chemotherapeutic regimens including Doxorubicin, Carboplatin, Cyclophosphamide or Doxetaxel could be used to target WT1 [40]. Most likely, due to the complexity of pathways and number of genes involved, combination therapy should be tested preclinically.

Our findings are not without limitations because of a small sample size; however, our study uncovered an intriguing survival advantage of *IDH* wild type chondrosarcomas. The analysis of larger patient cohorts may uncover unique characteristics of *IDH2* mutant tumors that our analysis was not able to capture. In addition, work in the future may be improved by better methods of extracting high-quality RNA from FFPE samples with chondroid matrix present. These methods may decrease the sample extraction batch effect that impacted our bioinformatic analysis.

## 5. Conclusions

In summary, dedifferentiated and higher-grade chondrosarcomas demonstrate genetic and probable epigenetic changes attributed in part to *IDH* status. The mutant and wild-type tumors utilize different molecular pathways which likely correlate with malignant behavior. In our admittedly small series, clinical outcomes are significantly different between *IDH* wild type and mutant groups. The combination of *IDH* mutated and dedifferentiated chondrosarcomas demonstrates the worst prognosis. However, dedifferentiated wild type tumors confer a better prognosis, which could be used in counseling patients. Future studies should explore whether targeting specific *IDH* mutations can be used to effectively inform therapeutic strategies for this aggressive disease with few options besides surgery.

## Figures and Tables

**Table 1:** Demographics of Grade 2, 3 and dedifferentiated chondrosarcomas with site, size and *IDH* status (- indicates data not available).

						*IDH*
Case	Age	Sex	Site	Size cm	Grade	Status
1	37	M	Arm	22.5	dd	Mut
2	81	M	Prox femur	17.6	dd	Mut
3	82	F	Thorax	24.1	2	WT
4	57	M	Pelvis	9.8	3	Mut
5	70	M	Sternum	-	2	WT
6	85	F	Humerus	-	dd	Mut
7	72	M	Femur	-	dd	Mut
8	83	F	Humerus	7.2	dd	-
9	77	M	Humerus	9.2	dd	Mut
10	56	M	Femur	-	2	Mut
			Scapula			
11	62	F	Chest	5.8	dd	Mut
12	44	M	Prox femur	21.2	dd	Mut
13	76	M	Chest wall	12.5	3	WT
14	65	M	Pelvis	6.3	3	-
15	76	M	Pelvis	-	2	Mut
16	91	M	Chest wall	10	dd	WT
17	58	M	Femur	-	2	Mut
18	38	M	Neck	-	3	Mut
19	56	F	Femur	33	dd	Mut
20	74	M	Pelvis	13.2	dd	WT
21	61	F	Pelvis	-	3	Mut
22	84	F	Hand	-	2	Mut
23	54	F	Femur	9	2	-
24	79	M	Prox femur	17.5	dd	Mut
25	70	F	Femur	-	2	Mut
26	72	F	Rib	7	2	WT
27	62	M	Pelvis	9	dd	Mut
28	72	F	Pelvis	13.5	2	-
29	67	M	Femur	27	dd	WT
30	74	M	Sacrum	6.2	dd	-
31	57	M	Humerus	9.7	2	-
32	71	M	Chest wall	4.5	2	-
33	88	F	Humerus	-	2	Mut
34	14	F	Pelvis	6	2	-
35	62	F	Chest wall	-	2	-
36	83	F	Femur	6	3	Mut
37	80	M	Pelvis	-	dd	WT
38	63	F	Talus	11	dd	-
39	70	M	Humerus	7	dd	-
40	18	F	Femur	-	2	-
41	74	M	Chest wall	2	2	-
42	83	M	Rib	6	dd	WT
43	88	M	Pelvis	5.1	2	-
44	64	M	Pelvis	10.6	dd	Mut
45	65	F	Femur	16	dd	-
46	66	M	Sternum	8	2	-
47	70	F	Humerus	-	dd	Mut
48	50	M	Femur	5.5	2	Mut
49	88	F	Femur	11	dd	Mut
50	51	M	Scapula	-	2	WT
51	34	F	Humerus	5.5	2	-
52	49	F	Thigh	-	3	-
53	43	M	Pelvis	25	2	WT
54	74	F	Femur	-	dd	WT
55	56	M	Pelvis	18	2	-
56	84	M	Pelvis	9	2	Mut
57	64	M	Femur	11.5	dd	WT
58	82	F	Femur	3.5	2	WT
59	68	M	Pelvis	5.5	2	Mut
60	70	M	Tibia	9.5	dd	WT
61	67	F	Humerus	14	dd	Mut
62	56	M	Femur	10	dd	Mut
63	71	F	Femur	20.5	dd	Mut
64	32	M	Pelvis	18	dd	Mut
65	74	F	Femur	6	dd	-
66	49	M	Pelvis	10.5	dd	-
67	75	M	Humerus	9.5	dd	-
68	57	M	Pelvis	12	dd	Mut
69	56	F	Pelvis	-	dd	Mut

Abbreviations: dd, dedifferentiated; Mut, *IDH* mutant; WT, wild type; Prox, proximal.

**Table 2:** Selected differentially expressed genes in *IDH* WT vs. mutant chondrosarcomas (grades 2, 3 and dedifferentiated).

DEG Higher in IDH WT	DEG Higher in IDH Mut
GREM1	COL10A1
TMEM52	MMP13
FOXA1	HHIP
ALX Homeobox 1, 3	IBSP
HOXA2	COL26A1
WT1	WRAP53
BMP7	TP53
SOX2	
IGF2	
MAGEA1	
NES	
ERG	
FGF18	
DKK2	

**Table 3:** Relative expression of genes of interest (compared to housekeeping genes) in *IDH* mutant fresh frozen chondrosarcomas using qPCR.

Case	COL10A1	MMP13	SATB2	IDH Mutation	Grade
9	Expressed	Expressed	Expressed	IDH1 R132S	Dedifferentiated
6	Expressed	Low Expression	Expressed	IDH2 R172S	Dedifferentiated
17	Expressed	Expressed	Expressed	IDH1 R132L	2
4	High Expression	High Expression	High Expression	IDH1 R132C	3
2	High Expression	High Expression	High Expression	IDH1 R132C	Dedifferentiated

## Data Availability

Data are contained within the article and Supplementary Materials.

## Supplementary Materials

The following supporting information can be downloaded at: https://www.mdpi.com/article/10.3390/cancers16020247/s1, Data S1: Upregulated IDH Differentially Expressed Genes; Data S2: WT Upregulated Differentially Expressed Genes; Data S3: Heatmap of IDH mutant and WT chondrosarcomas with SATB2; Data S4: Heatmap of IDH mutant and WT CS with ARL4C; Webpage Details for gene set CTCTATG_MIR368 (C3 GSEA with SATB2); Webpage Details for gene set KAZMIN PBM (C7 GSEA with ARL4C).

## References

[1] WHO Classification of Tumours Editorial Board (2020). Soft tissue and bone tumours. WHO Classification of Tumours Series.

[2] Nakagawa M., Sekimizu M., Endo M., Kobayashi E., Iwata S., Fukushima S., Yoshida A., Kitabayashi I., Ichikawa H., Kawai A. (2022). Prognostic impact of IDH mutations in chondrosarcoma. J. Orthop. Sci..

[3] Micaily I., Roche M., Ibrahim M., Martinez-Outschoorn U., Mallick A. (2021). Metabolic pathways and targets in chondrosarcoma. Front. Oncol..

[4] Zhang H., Puviindran V., Puviindran N., Ding X., Shen L., Tang Y., Tsushima H., Yahara Y., Ban G., Zhang G. (2022). Distinct roles of glutamine metabolism in benign and malignant cartilage tumors with IDH mutations. J. Bone Miner. Res..

[5] Cross W., Lyskjaer I., Lesluyes T., Hargreaves S., Strobl A., Davies C., Waise S., Hames-Fathi S., Oukrif D., Ye H. (2022). A genetic model for central chondrosarcoma evolution correlates with patient outcomes. Genome Med..

[6] Miwa S., Yamamoto N., Hayashi K., Takeuchi A., Igarashi K., Tsuchiya H. (2022). Therapeutic targets and emerging treatments in advanced chondrosarcoma. Int. J. Mol. Sci..

[7] Cairns R., Mak T. (2013). Oncogenic isocitrate dehydrogenase mutations: Mechanisms, models and clinical opportunities. Cancer Discov..

[8] Molenaar R., Wilmink J. (2022). IDH1/2 mutations in cancer stem cells and their implications for differentiation therapy. J. Histochem. Cytochem..

[9] Bovee J., Cleton-Jansen A., Rosenberg C., Taminiau A., Cornelisse C.J., Hogendoorn P. (1999). Molecular genetic characterization of both components of a dedifferentiated chondrosarcoma, with implications for its histogenesis. J. Pathol..

[10] Nielsen G., Rosenberg A. (2017). Diagnostic Pathology Bone.

[11] Tap W., Villalobos V., Cole G., Burris H., Janku F., Mir O., Beeram M., Wagner A., Jiang L., Wu B. (2020). Phase 1 study of the mutant IDH1 inhibitor Ivosidenib: Safety and clinical activity in patients with advanced chondrosarcoma. J. Clin. Oncol..

[12] National Comprehensive Cancer Network (2023). NCCN Clinical Practice Guidelines in Oncology, Bone Cancer, Version 1.2024.

[13] Mitchell A., Ayoub K., Mangham D., Grimer R., Carter S., Tillman R. (2000). Experience in the treatment of dedifferentiated chondrosarcoma. J. Bone Jt. Surg. Br..

[14] Italiano A., Mir O., Cioffi A., Palmerini E., Piperno-Neumann S., Perrin C., Chaigneau L., Penel N., Duffaud F., Kurtz J. (2013). Advanced chondrosarcomas: Role of chemotherapy and survival. Ann. Oncol..

[15] Schuetze S., Bolejack V., Choy E., Ganjoo K., Staddon A., Chow W., Tawbi H., Samuels B., Patel S., von Mehren M. (2017). Phase 2 study of dasatinib in patients with alveolar soft part sarcoma, chondrosarcoma, chordoma, epithelioid sarcoma, or solitary fibrous tumor. Cancer.

[16] Chow W., Frankel P., Ruel C., Araujo D., Milhem M., Okuno S., Hartner L., Undevia S., Staddon A. (2020). Results of a prospective phase 2 study of pazopanib in patients with surgically unresectable or metastatic chondrosarcoma. Cancer.

[17] Horbinski C., Kofler J., Kelly L., Murdoch G., Nikiforova M. (2009). Diagnostic use of IDH1/2 mutation analysis in routine clinical testing of formalin fixed, paraffin embedded glioma tissues. J. Neuropathol. Exp. Neurol..

[18] Pirozzi C., Yan H. (2021). The implications of IDH mutations for cancer development and therapy. Nat. Rev. Clin. Oncol..

[19] Han S., Liu Y., Cai S., Qian M., Ding J., Larion M., Gilbert M., Yang C. (2020). IDH mutation in glioma: Molecular mechanisms and potential therapeutic targets. Br. J. Cancer.

[20] WHO Classification of Tumours Editorial Board (2021). Central Nervous System Tumours. WHO Classification of Tumours Series.

[21] Tian W., Zhang W., Wang Y., Jin R., Wang Y., Guo H., Tang Y., Yao X. (2022). Recent advances in IDH1 mutant inhibitor in cancer therapy. Front. Pharmacol..

[22] Nacev B., Sanchez-Vega F., Smith S., Antonescu B., Rosenbaum E., Shi H., Tang C., Socci N., Rana S., Gularte-Merida R. (2022). Clinical sequencing of soft tissue and bone sarcomas delineates diverse genomic landscapes and potential therapeutic targets. Nat. Commun..

[23] Guo C., Pirozzi C., Lopez G., Yan H. (2011). Isocitrate dehydrogenase mutations in gliomas: Mechanisms, biomarkers and therapeutic target. Curr. Opin. Neurol..

[24] Nicolle R., Ayadi M., Gomez-Brouchet A., Armenoult L., Banneau G., Elarouci N., Tallegas M., Decouvelaere A., Aubert S., Redini F. (2019). Integrated molecular characterization of chondrosarcoma reveals critical determinants of disease progression. Nat. Commun..

[25] Venneker S., Kruisselbrink A., Baranski Z., Palubeckaite I., Briaire-de Bruijn I., Oosting J., French P., Danen E., Bovee J. (2020). Beyond the influence of IDH mutations: Exploring epigenetic vulnerabilities in chondrosarcoma. Cancers.

[26] Amary M., Bacsi K., Maggiani F., Damato S., Halai D., Berisha F., Pollock R., O’Donnell P., Grigoriadis A., Diss T. (2011). IDH1 and IDH2 mutations are frequent events in central chondrosarcoma and central and periosteal chondromas but not in other mesenchymal tumours. J. Pathol..

[27] Mohammad N., Wong D., Lum A., Lin J., Ho J., Lee C., Yip S. (2020). Characterization of isocitrate dehydrogenase 1/isocitrate dehydrogenase 2 gene mutation and the d-2-hydroxyglutarate oncometabolite level in dedifferentiated chondrosarcoma. Histopathology.

[28] Stoeckl S., Lindner G., Li S., Schuster P., Haferkamp S., Wagner F., Prodinger P., Multhoff G., Boxberg M., Hillman A. (2020). SOX9 knockout induces polyploidy and changes sensitivity to tumor treatment strategies in a chondrosarcoma cell line. Int. J. Mol. Sci..

[29] Blumer M. (2021). Bone tissue and histological and molecular events during development of the long bones. Ann. Anat..

[30] Iseulys R., Gomez-Brouchet A., Bouvier C., Du Bouexic G., Karanian M., Blay J., Dutour A. (2020). The immune landscape of chondrosarcoma reveals an immunosuppressive environment in the dedifferentiated subtypes and exposes CSFR1 + macrophages as a promising therapeutic target. J. Bone Oncol..

[31] Tiet T., Hopyan S., Nadesan P., Gokgoz N., Poon R., Lin A., Yan T., Andrulis I., Alman B., Wunder J. (2006). Constitutive hedgehog signaling in chondrosarcoma up-regulates tumor cell proliferation. Am. J. Pathol..

[32] Meijer D., de Jong D., Pansuriya T.C., van den Akker B., Picci P., Szuhai K., Bovee J. (2012). Genetic characterization of mesenchymal, clear cell and dedifferentiated chondrosarcoma. Genes Chromosomes Cancer.

[33] Gadelha R., Machado C., de Pinho Pessoa F., Pantoja L., Barreto I., Ribeiro R., de Moraes Filho M., de Moraes M., Khayat A., Moreira-Nunes C. (2022). The role of WRAP53 in cell homeostasis and carcinogenesis onset. Curr. Issues Mol. Biol..

[34] Takahashi K., Tanabe K., Ohnuki M., Narita M., Ichisaka T., Tomoda K., Yamanaka S. (2007). Induction of pluripotent stem cells from adult human fibroblasts by defined factors. Cell.

[35] Liu Z., Shen F., Wang H., Li A., Wang J., Du L., Liu B., Zhang B., Lian X., Pang B. (2020). Abnormally high expression of HOXA2 as an independent factor for poor prognosis in glioma patients. Cell Cycle.

[36] De Pagter-Holthuizen P., Jansen M., van der Kammen R., van Schaik F., Sussenback J. (1988). Differential expression of the human insulin-like growth factor II gene. Characterization of the IGF-II mRNAs and an mRNA encoding a putative IGF-II associated protein. Biochem. Biophys. Acta.

[37] Palmini G., Marini F., Brandi M. (2017). What is new in the miRNA world regarding osteosarcoma and chondrosarcoma?. Molecules.

[38] Florke Gee R., Chen H., Lee A., Daly C., Wilander B., Tacer K., Potts P. (2020). Emerging roles of the MAGE protein family in stress response pathways. J. Biol. Chem..

[39] Kimura K., Matsumoto S., Harada T., Morii E., Nagatomo I., Shintani Y., Kikuchi A. (2020). ARL4C is associated with initiation and progression of lung adenocarcinoma and represents a therapeutic target. Cancer Sci..

[40] www.genecards.org.

