# Supplementary material for: Grade 2, 3 and Dedifferentiated Chondrosarcomas: A Comparative Study of Isocitrate Dehydrogenase-Mutant and Wild-Type Tumors with Implications for Prognosis and Therapy
Source: Cancers (Basel). 2024 Jan 5;16(2):247. doi: 10.3390/cancers16020247 (PMC10813891; doi:10.3390/cancers16020247)
Supplement: Supplementary file 1 [file cancers-16-00247-s001.zip › Details for gene set CTCTATG_MIR368[GSEA] (5).htm]

Details for gene set CTCTATG\_MIR368[GSEA]

|  |  |
| --- | --- |
| Dataset | Master\_CPMs\_collapsed\_to\_symbols.WT\_vs\_IDH.cls#WT\_versus\_IDH |
| Phenotype | WT\_vs\_IDH.cls#WT\_versus\_IDH |
| Upregulated in class | IDH |
| GeneSet | CTCTATG\_MIR368 |
| Enrichment Score (ES) | -0.39315525 |
| Normalized Enrichment Score (NES) | -1.2545276 |
| Nominal p-value | 0.14145383 |
| FDR q-value | 1.0 |
| FWER p-Value | 0.997 |
Table: GSEA Results Summary

  

Fig 1: Enrichment plot: CTCTATG\_MIR368      
 Profile of the Running ES Score & Positions of GeneSet Members on the Rank Ordered List

  

|  | SYMBOL | TITLE | RANK IN GENE LIST | RANK METRIC SCORE | RUNNING ES | CORE ENRICHMENT |
| --- | --- | --- | --- | --- | --- | --- |
| 1 | NFIB | nuclear factor I B [Source:HGNC Symbol;Acc:HGNC:7785] | 794 | 0.712 | 0.0437 | No |
| 2 | CAPN3 | calpain 3 [Source:HGNC Symbol;Acc:HGNC:1480] | 1145 | 0.651 | 0.0928 | No |
| 3 | SMARCA2 | SWI/SNF related, matrix associated, actin dependent regulator of chromatin, subfamily a, member 2 [Source:HGNC Symbol;Acc:HGNC:11098] | 4348 | 0.431 | 0.0527 | No |
| 4 | CACNA1G | calcium voltage-gated channel subunit alpha1 G [Source:HGNC Symbol;Acc:HGNC:1394] | 6159 | 0.371 | 0.0414 | No |
| 5 | PAX2 | paired box 2 [Source:HGNC Symbol;Acc:HGNC:8616] | 10701 | 0.238 | -0.0484 | No |
| 6 | RGS4 | regulator of G protein signaling 4 [Source:HGNC Symbol;Acc:HGNC:10000] | 11203 | 0.223 | -0.0408 | No |
| 7 | DLX1 | distal-less homeobox 1 [Source:HGNC Symbol;Acc:HGNC:2914] | 11756 | 0.214 | -0.0353 | No |
| 8 | SEPTIN7 | septin 7 [Source:HGNC Symbol;Acc:HGNC:1717] | 11769 | 0.214 | -0.0167 | No |
| 9 | XKR6 | XK related 6 [Source:HGNC Symbol;Acc:HGNC:27806] | 11888 | 0.211 | -0.0009 | No |
| 10 | BDNF | brain derived neurotrophic factor [Source:HGNC Symbol;Acc:HGNC:1033] | 12023 | 0.206 | 0.0141 | No |
| 11 | TM2D1 | TM2 domain containing 1 [Source:HGNC Symbol;Acc:HGNC:24142] | 13084 | 0.179 | 0.0041 | No |
| 12 | ASXL1 | ASXL transcriptional regulator 1 [Source:HGNC Symbol;Acc:HGNC:18318] | 13447 | 0.170 | 0.0103 | No |
| 13 | PARD3B | par-3 family cell polarity regulator beta [Source:HGNC Symbol;Acc:HGNC:14446] | 15078 | 0.127 | -0.0183 | No |
| 14 | CABLES1 | Cdk5 and Abl enzyme substrate 1 [Source:HGNC Symbol;Acc:HGNC:25097] | 15798 | 0.110 | -0.0261 | No |
| 15 | ZNF146 | zinc finger protein 146 [Source:HGNC Symbol;Acc:HGNC:12931] | 16329 | 0.100 | -0.0302 | No |
| 16 | SRSF3 | serine and arginine rich splicing factor 3 [Source:HGNC Symbol;Acc:HGNC:10785] | 16495 | 0.094 | -0.0259 | No |
| 17 | FEM1C | fem-1 homolog C [Source:HGNC Symbol;Acc:HGNC:16933] | 17294 | 0.075 | -0.0388 | No |
| 18 | CSTF3 | cleavage stimulation factor subunit 3 [Source:HGNC Symbol;Acc:HGNC:2485] | 18243 | 0.061 | -0.0566 | No |
| 19 | CNIH1 | cornichon family AMPA receptor auxiliary protein 1 [Source:HGNC Symbol;Acc:HGNC:19431] | 18741 | 0.046 | -0.0646 | No |
| 20 | DACH1 | dachshund family transcription factor 1 [Source:HGNC Symbol;Acc:HGNC:2663] | 19643 | 0.020 | -0.0849 | No |
| 21 | EYA4 | EYA transcriptional coactivator and phosphatase 4 [Source:HGNC Symbol;Acc:HGNC:3522] | 27068 | -0.022 | -0.2643 | No |
| 22 | FBXL3 | F-box and leucine rich repeat protein 3 [Source:HGNC Symbol;Acc:HGNC:13599] | 27405 | -0.029 | -0.2699 | No |
| 23 | GRB2 | growth factor receptor bound protein 2 [Source:HGNC Symbol;Acc:HGNC:4566] | 27659 | -0.034 | -0.2731 | No |
| 24 | AFF4 | AF4/FMR2 family member 4 [Source:HGNC Symbol;Acc:HGNC:17869] | 30949 | -0.116 | -0.3431 | No |
| 25 | MEF2C | myocyte enhancer factor 2C [Source:HGNC Symbol;Acc:HGNC:6996] | 31911 | -0.144 | -0.3538 | No |
| 26 | VPS54 | VPS54 subunit of GARP complex [Source:HGNC Symbol;Acc:HGNC:18652] | 33356 | -0.181 | -0.3730 | No |
| 27 | VHLL | VHL like [Source:HGNC Symbol;Acc:HGNC:30666] | 34180 | -0.206 | -0.3749 | Yes |
| 28 | MMP25 | matrix metallopeptidase 25 [Source:HGNC Symbol;Acc:HGNC:14246] | 34430 | -0.215 | -0.3620 | Yes |
| 29 | FGF7P6 | fibroblast growth factor 7 pseudogene 6 [Source:HGNC Symbol;Acc:HGNC:27852] | 34833 | -0.227 | -0.3517 | Yes |
| 30 | SET | SET nuclear proto-oncogene [Source:HGNC Symbol;Acc:HGNC:10760] | 34993 | -0.231 | -0.3350 | Yes |
| 31 | TRNP1 | TMF1 regulated nuclear protein 1 [Source:HGNC Symbol;Acc:HGNC:34348] | 35879 | -0.265 | -0.3332 | Yes |
| 32 | PTGES2 | prostaglandin E synthase 2 [Source:HGNC Symbol;Acc:HGNC:17822] | 37005 | -0.308 | -0.3333 | Yes |
| 33 | SGMS1 | sphingomyelin synthase 1 [Source:HGNC Symbol;Acc:HGNC:29799] | 39028 | -0.424 | -0.3452 | Yes |
| 34 | MMP19 | matrix metallopeptidase 19 [Source:HGNC Symbol;Acc:HGNC:7165] | 39156 | -0.433 | -0.3099 | Yes |
| 35 | MYLK | myosin light chain kinase [Source:HGNC Symbol;Acc:HGNC:7590] | 40095 | -0.554 | -0.2837 | Yes |
| 36 | ZDHHC1 | zinc finger DHHC-type containing 1 [Source:HGNC Symbol;Acc:HGNC:17916] | 40485 | -0.642 | -0.2363 | Yes |
| 37 | RASAL2 | RAS protein activator like 2 [Source:HGNC Symbol;Acc:HGNC:9874] | 40527 | -0.654 | -0.1794 | Yes |
| 38 | RNF11 | ring finger protein 11 [Source:HGNC Symbol;Acc:HGNC:10056] | 40672 | -0.702 | -0.1207 | Yes |
| 39 | UTS2 | urotensin 2 [Source:HGNC Symbol;Acc:HGNC:12636] | 40673 | -0.702 | -0.0586 | Yes |
| 40 | SATB2 | SATB homeobox 2 [Source:HGNC Symbol;Acc:HGNC:21637] | 40751 | -0.743 | 0.0054 | Yes |
Table: GSEA details [plain text format]

  

Fig 2: CTCTATG\_MIR368      
 Blue-Pink O' Gram in the Space of the Analyzed GeneSet

  

Fig 3: CTCTATG\_MIR368: Random ES distribution      
 Gene set null distribution of ES for **CTCTATG\_MIR368**

  
