# Supplementary material for: Grade 2, 3 and Dedifferentiated Chondrosarcomas: A Comparative Study of Isocitrate Dehydrogenase-Mutant and Wild-Type Tumors with Implications for Prognosis and Therapy
Source: Cancers (Basel). 2024 Jan 5;16(2):247. doi: 10.3390/cancers16020247 (PMC10813891; doi:10.3390/cancers16020247)
Supplement: Supplementary file 1 [file cancers-16-00247-s001.zip › Details for gene set KAZMIN_PBMC_P_FALCIPARUM_RTSS_AS01_AGE_UNKNOWN_CORRELATED_WITH_PROTECTION_56DY_NEGATIVE[GSEA].html]

Details for gene set KAZMIN\_PBMC\_P\_FALCIPARUM\_RTSS\_AS01\_AGE\_UNKNOWN\_CORRELATED\_WITH\_PROTECTION\_56DY\_NEGATIVE[GSEA]

|  |  |
| --- | --- |
| Dataset | Master\_CPMs\_collapsed\_to\_symbols.WT\_vs\_IDH.cls#WT\_versus\_IDH |
| Phenotype | WT\_vs\_IDH.cls#WT\_versus\_IDH |
| Upregulated in class | WT |
| GeneSet | KAZMIN\_PBMC\_P\_FALCIPARUM\_RTSS\_AS01\_AGE\_UNKNOWN\_CORRELATED\_WITH\_PROTECTION\_56DY\_NEGATIVE |
| Enrichment Score (ES) | 0.5261099 |
| Normalized Enrichment Score (NES) | 1.5288353 |
| Nominal p-value | 0.028985508 |
| FDR q-value | 1.0 |
| FWER p-Value | 0.757 |
Table: GSEA Results Summary

  

Fig 1: Enrichment plot: KAZMIN\_PBMC\_P\_FALCIPARUM\_RTSS\_AS01\_AGE\_UNKNOWN\_CORRELATED\_WITH\_PROTECTION\_56DY\_NEGATIVE      
 Profile of the Running ES Score & Positions of GeneSet Members on the Rank Ordered List

  

|  | SYMBOL | TITLE | RANK IN GENE LIST | RANK METRIC SCORE | RUNNING ES | CORE ENRICHMENT |
| --- | --- | --- | --- | --- | --- | --- |
| 1 | GZMM | granzyme M [Source:HGNC Symbol;Acc:HGNC:4712] | 72 | 1.043 | 0.0709 | Yes |
| 2 | PRKCQ | protein kinase C theta [Source:HGNC Symbol;Acc:HGNC:9410] | 160 | 0.931 | 0.1336 | Yes |
| 3 | TBX21 | T-box transcription factor 21 [Source:HGNC Symbol;Acc:HGNC:11599] | 344 | 0.827 | 0.1867 | Yes |
| 4 | CD244 | CD244 molecule [Source:HGNC Symbol;Acc:HGNC:18171] | 585 | 0.763 | 0.2339 | Yes |
| 5 | GZMB | granzyme B [Source:HGNC Symbol;Acc:HGNC:4709] | 1020 | 0.671 | 0.2700 | Yes |
| 6 | CD7 | CD7 molecule [Source:HGNC Symbol;Acc:HGNC:1695] | 1225 | 0.643 | 0.3098 | Yes |
| 7 | CST7 | cystatin F [Source:HGNC Symbol;Acc:HGNC:2479] | 1597 | 0.604 | 0.3428 | Yes |
| 8 | ZAP70 | zeta chain of T cell receptor associated protein kinase 70 [Source:HGNC Symbol;Acc:HGNC:12858] | 1613 | 0.602 | 0.3844 | Yes |
| 9 | IL18RAP | interleukin 18 receptor accessory protein [Source:HGNC Symbol;Acc:HGNC:5989] | 1967 | 0.573 | 0.4157 | Yes |
| 10 | GIMAP7 | GTPase, IMAP family member 7 [Source:HGNC Symbol;Acc:HGNC:22404] | 2741 | 0.518 | 0.4329 | Yes |
| 11 | GNLY | granulysin [Source:HGNC Symbol;Acc:HGNC:4414] | 2949 | 0.505 | 0.4630 | Yes |
| 12 | CD96 | CD96 molecule [Source:HGNC Symbol;Acc:HGNC:16892] | 3462 | 0.477 | 0.4837 | Yes |
| 13 | KLRD1 | killer cell lectin like receptor D1 [Source:HGNC Symbol;Acc:HGNC:6378] | 4259 | 0.435 | 0.4945 | Yes |
| 14 | ADGRG1 | adhesion G protein-coupled receptor G1 [Source:HGNC Symbol;Acc:HGNC:4512] | 4745 | 0.415 | 0.5116 | Yes |
| 15 | KLRF1 | killer cell lectin like receptor F1 [Source:HGNC Symbol;Acc:HGNC:13342] | 5287 | 0.398 | 0.5261 | Yes |
| 16 | KLRB1 | killer cell lectin like receptor B1 [Source:HGNC Symbol;Acc:HGNC:6373] | 6625 | 0.357 | 0.5183 | No |
| 17 | KIR3DL3 | killer cell immunoglobulin like receptor, three Ig domains and long cytoplasmic tail 3 [Source:HGNC Symbol;Acc:HGNC:16312] | 7815 | 0.320 | 0.5115 | No |
| 18 | KIR3DL1 | killer cell immunoglobulin like receptor, three Ig domains and long cytoplasmic tail 1 [Source:HGNC Symbol;Acc:HGNC:6338] | 9715 | 0.267 | 0.4837 | No |
| 19 | HOPX | HOP homeobox [Source:HGNC Symbol;Acc:HGNC:24961] | 10630 | 0.240 | 0.4781 | No |
| 20 | KIR2DL1 | killer cell immunoglobulin like receptor, two Ig domains and long cytoplasmic tail 1 [Source:HGNC Symbol;Acc:HGNC:6329] | 11298 | 0.223 | 0.4773 | No |
| 21 | GZMH | granzyme H [Source:HGNC Symbol;Acc:HGNC:4710] | 11990 | 0.207 | 0.4749 | No |
| 22 | NKG7 | natural killer cell granule protein 7 [Source:HGNC Symbol;Acc:HGNC:7830] | 12166 | 0.203 | 0.4847 | No |
| 23 | KLRC1 | killer cell lectin like receptor C1 [Source:HGNC Symbol;Acc:HGNC:6374] | 15823 | 0.109 | 0.4030 | No |
| 24 | S1PR5 | sphingosine-1-phosphate receptor 5 [Source:HGNC Symbol;Acc:HGNC:14299] | 15948 | 0.105 | 0.4073 | No |
| 25 | PVRIG | PVR related immunoglobulin domain containing [Source:HGNC Symbol;Acc:HGNC:32190] | 18087 | 0.063 | 0.3594 | No |
| 26 | SAMD3 | sterile alpha motif domain containing 3 [Source:HGNC Symbol;Acc:HGNC:21574] | 18225 | 0.061 | 0.3603 | No |
| 27 | GZMA | granzyme A [Source:HGNC Symbol;Acc:HGNC:4708] | 18298 | 0.059 | 0.3627 | No |
| 28 | CD247 | CD247 molecule [Source:HGNC Symbol;Acc:HGNC:1677] | 19588 | 0.022 | 0.3327 | No |
| 29 | KIR2DL3 | killer cell immunoglobulin like receptor, two Ig domains and long cytoplasmic tail 3 [Source:HGNC Symbol;Acc:HGNC:6331] | 23179 | 0.000 | 0.2450 | No |
| 30 | STAT4 | signal transducer and activator of transcription 4 [Source:HGNC Symbol;Acc:HGNC:11365] | 27543 | -0.033 | 0.1407 | No |
| 31 | XCL2 | X-C motif chemokine ligand 2 [Source:HGNC Symbol;Acc:HGNC:10646] | 27722 | -0.036 | 0.1389 | No |
| 32 | CLIC3 | chloride intracellular channel 3 [Source:HGNC Symbol;Acc:HGNC:2064] | 28792 | -0.057 | 0.1167 | No |
| 33 | TGFBR3 | transforming growth factor beta receptor 3 [Source:HGNC Symbol;Acc:HGNC:11774] | 28915 | -0.060 | 0.1179 | No |
| 34 | PRF1 | perforin 1 [Source:HGNC Symbol;Acc:HGNC:9360] | 29004 | -0.063 | 0.1201 | No |
| 35 | KLRK1 | killer cell lectin like receptor K1 [Source:HGNC Symbol;Acc:HGNC:18788] | 29049 | -0.064 | 0.1235 | No |
| 36 | MYBL1 | MYB proto-oncogene like 1 [Source:HGNC Symbol;Acc:HGNC:7547] | 29469 | -0.075 | 0.1185 | No |
| 37 | PTGDR | prostaglandin D2 receptor [Source:HGNC Symbol;Acc:HGNC:9591] | 30106 | -0.092 | 0.1094 | No |
| 38 | CCL5 | C-C motif chemokine ligand 5 [Source:HGNC Symbol;Acc:HGNC:10632] | 30146 | -0.094 | 0.1149 | No |
| 39 | RORA | RAR related orphan receptor A [Source:HGNC Symbol;Acc:HGNC:10258] | 33808 | -0.196 | 0.0391 | No |
| 40 | SH2D2A | SH2 domain containing 2A [Source:HGNC Symbol;Acc:HGNC:10821] | 34517 | -0.218 | 0.0370 | No |
| 41 | IL2RB | interleukin 2 receptor subunit beta [Source:HGNC Symbol;Acc:HGNC:6009] | 38512 | -0.381 | -0.0340 | No |
| 42 | KIR2DL4 | killer cell immunoglobulin like receptor, two Ig domains and long cytoplasmic tail 4 [Source:HGNC Symbol;Acc:HGNC:6332] | 38738 | -0.399 | -0.0117 | No |
| 43 | ARL4C | ADP ribosylation factor like GTPase 4C [Source:HGNC Symbol;Acc:HGNC:698] | 40930 | -0.952 | 0.0010 | No |
Table: GSEA details [plain text format]

  

Fig 2: KAZMIN\_PBMC\_P\_FALCIPARUM\_RTSS\_AS01\_AGE\_UNKNOWN\_CORRELATED\_WITH\_PROTECTION\_56DY\_NEGATIVE      
 Blue-Pink O' Gram in the Space of the Analyzed GeneSet

  

Fig 3: KAZMIN\_PBMC\_P\_FALCIPARUM\_RTSS\_AS01\_AGE\_UNKNOWN\_CORRELATED\_WITH\_PROTECTION\_56DY\_NEGATIVE: Random ES distribution      
 Gene set null distribution of ES for **KAZMIN\_PBMC\_P\_FALCIPARUM\_RTSS\_AS01\_AGE\_UNKNOWN\_CORRELATED\_WITH\_PROTECTION\_56DY\_NEGATIVE**

  
