# Supplementary figures and images for: Grade 2, 3 and Dedifferentiated Chondrosarcomas: A Comparative Study of Isocitrate Dehydrogenase-Mutant and Wild-Type Tumors with Implications for Prognosis and Therapy
Source: Cancers (Basel). 2024 Jan 5;16(2):247. doi: 10.3390/cancers16020247 (PMC10813891; doi:10.3390/cancers16020247)

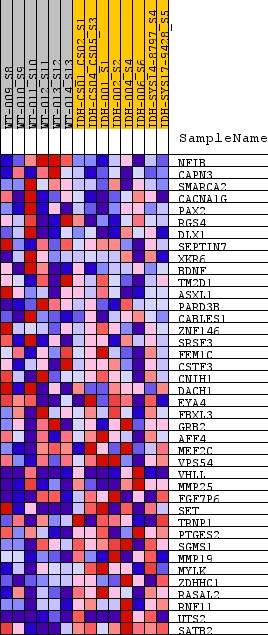

Supplement: Supplementary file 1 [file cancers-16-00247-s001.zip › Data S3 Heatmap of IDHMutant and WT CS with SATB2 CTCTATG_MIR368_106.jpg]

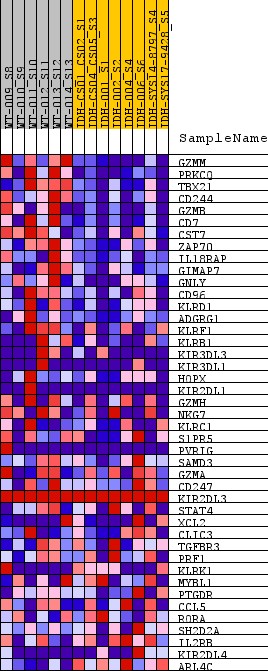

Supplement: Supplementary file 1 [file cancers-16-00247-s001.zip › DataS4 Heatmap of IDHmutant and WT CS KAZMIN with ARL4C.jpg]
